# Cellulosic ethanol: interactions between cultivar and enzyme loading in wheat straw processing

**DOI:** 10.1186/1754-6834-3-25

**Published:** 2010-11-18

**Authors:** Jane Lindedam, Sander Bruun, Henning Jørgensen, Claus Felby, Jakob Magid

**Affiliations:** 1 Plant and Soil Science Laboratory, Department of Agriculture and Ecology, Faculty of Life Sciences, University of Copenhagen, Denmark; 2 Forestry and Wood Products, Forest & Landscape, Faculty of Life Sciences, University of Copenhagen, Denmark

## Abstract

**Background:**

Variations in sugar yield due to genotypic qualities of feedstock are largely undescribed for pilot-scale ethanol processing. Our objectives were to compare glucose and xylose yield (conversion and total sugar yield) from straw of five winter wheat cultivars at three enzyme loadings (2.5, 5 and 10 FPU g^-1 ^dm pretreated straw) and to compare particle size distribution of cultivars after pilot-scale hydrothermal pretreatment.

**Results:**

Significant interactions between enzyme loading and cultivars show that breeding for cultivars with high sugar yields under modest enzyme loading could be warranted. At an enzyme loading of 5 FPU g^-1 ^dm pretreated straw, a significant difference in sugar yields of 17% was found between the highest and lowest yielding cultivars. Sugar yield from separately hydrolyzed particle-size fractions of each cultivar showed that finer particles had 11% to 21% higher yields than coarse particles. The amount of coarse particles from the cultivar with lowest sugar yield was negatively correlated with sugar conversion.

**Conclusions:**

We conclude that genetic differences in sugar yield and response to enzyme loading exist for wheat straw at pilot scale, depending on differences in removal of hemicellulose, accumulation of ash and particle-size distribution introduced by the pretreatment.

## Background

Lignocellulosic biomass is commonly recognized as a potential sustainable source of mixed sugars for fermentation to biofuels. A challenge remains, however, to make the process of converting lignocellulosics to biofuels cost-competitive in a large-scale process [1]. One way of achieving cost reductions and yield increments for the conversion process could be attained through improving biofeedstock quality [2]. Ethanol yields from different cultivars have been found to vary with the cultivars. Examples are corn stover [3,4], grasses [5], winter triticale grain [6] and winter wheat straw [7]. These studies have all employed small-scale pretreatment and hydrolysis. Since small-scale assays do not fully reflect the conditions of running a full commercial-scale pretreatment and hydrolysis, it can be questioned whether results from the small-scale assays can be extrapolated to larger-scale plants.

Previous work shows wheat straw ethanol yields varying from 31% to 84% of theoretical maximum value, depending on the pretreatment method applied, enzyme loading during hydrolysis [8] and yeast culture used [9-12], as well as cultivar and local growing conditions which affect the composition of the biomass. As enzymes are costly and currently constitute one of the largest expenses in second-generation bioethanol production, the release of sugars at a given enzyme loading for different cultivars is of importance.

Release of fermentable sugars is affected by physical and chemical structural features as summarized by Chang and Holtzapple [13]. Diminution of substrate particles has previously been shown to increase sugar yield during enzymatic hydrolysis of lignocellulosic residues by increasing the surface available to enzymes and reducing the crystallinity of the sample [14]. We therefore hypothesize that particle-size distribution of processed biomass can be related to the sugar yield from different cultivars. Most previous studies of the relationship between sugar yield and substrate particle sizes have been conducted by grinding the biomass to the desired sizes or otherwise fractionating before hydrolysis, in which case larger particle-size fractions have been found to be more resistant to hydrolysis compared with smaller size fractions [15,16]. This will result in a distribution which is mainly a result of the milling and sieving processes and not the pretreatment process *per se*. We therefore decided to examine the particle-size distribution after pretreatment and the subsequent convertibility to fermentable sugars.

In this work, wheat cultivars are compared in terms of sugar yield and response to enzyme loadings and particle-size distribution after pretreatment in a large pilot-scale plant.

## Methods

### Raw materials

Wheat straw (*Triticum aestivum *L.) was grown and collected during harvest in June 2008 at two locations in Denmark: Fynen (55° 24' N, 10° 23' E) and Holstebro (56° 21' N, 8° 37' E). Straw from five cultivars was used: Ambition, Hereford, Skalmeje, Smuggler and Frument. These are typical winter wheat cultivars in northern Europe and made up more than 80% of the winter wheat seed sales in Denmark in 2008. Hereford, Frument and Ambition are agronomical high-yielding cultivars. Smuggler is characterized by slightly lower but more stable crop yields. Skalmeje is an older cultivar still in use, but rapidly leaving the market because of low crop yield performance. Skalmeje has been popular because of high straw stiffness and good pest resistance. Bales of each straw type, approximately 500 kg per bale, were collected and stored in a dry, nonheated room. At pilot-scale level, pretreatment and subsequent fractionation was performed on all bales, while tests with fractionation before bench-scale pretreatment and hydrolysis was performed on Ambition from Holstebro.

### Analytical methods

The composition of the untreated and pretreated straw was determined by two-step acid hydrolysis of the carbohydrates according to the procedure published by the National Renewable Energy Laboratory (NREL) [17]. Released sugars from acid hydrolysis and from the hydrolysates from all enzymatic hydrolysis were quantified on a Dionex Summit high-pressure liquid chromatography (HPLC) system (Dionex, Hvidovre, Denmark) equipped with a Shimadzu RI-detector (Shimadzu Europa GmbH, Germany). Separation was performed in a Phenomenex Rezex RHM column (Phenomenex, Alleroed, Denmark) at 80°C with 5 mM H_2_SO_4 _as eluent at a flow rate of 0.6 mL min^-1^. Samples were filtered through a 0.20-μm filter and diluted with eluent before analysis on HPLC.

The convertibility of each cultivar from cellulose and xylan was calculated as the amount of released glucose and xylose as a percentage of the maximum theoretical release as follows:

(1) $$\text{Convertibility }\left(x\right)=\frac{C_{xenz}(\text{g}/\text{l})}{C_{xcomposition}(\text{g}/\text{l})}*100\%,$$

where *x *denotes glucose, xylose or both (TS for total sugar), *C_x enz _*is the concentration of *x *measured after enzymatic hydrolysis and *C_x composition _*denotes the maximum possible concentration of *x*, calculated from compositional analysis of the fibers after pretreatment corrected for hydration by factors of 1.111 for measured glucan, 1.1362 for measured xylan and solid loading in the hydrolysis.

Sugar yield from each cultivar was calculated as a release of total sugar in grams per gram of dry matter of pretreated biomass (g g^-1 ^dm ptb). Sugar yield from particle-size fractions separated prior to pretreatment was based on dry matter nonpretreated biomass. Principal component analysis (PCA) and partial least squares (PLS) regression analysis were done in LatentiX 2.00 (Latent5, Copenhagen, Denmark, http://www.latentix.com/), and statistical evaluations were calculated using SAS software (SAS, Cary, North Carolina) with generalized linear models and mixed-effects models [18].

### Pretreatment at pilot-scale

During December 2008, one bale of each cultivar from each site was pretreated in Inbicon's pilot plant [19,20] in separate runs. Each bale was mechanically shredded to 5- to 10-cm pieces and fed continuously with a flow rate of 50 kg h^-1 ^to a soaking reactor, where it remained for 5-10 min at 80°C in 3 g L^-1 ^acetic acid solution. Excess water was removed, and straw was fed to the pretreatment reactor and moved through countercurrent fresh process water for 10 min at 195°C, severity index 3.8 [21]. Fibers were discharged continuously from the reactor with a dry matter content of 25-40%. After changing to a new cultivar, the system was operated until assumed steady state (2 h) before pretreated straw was sampled. The product was 10 different pretreated straw batches. Equal amounts of liquid were used in the pretreatment of each bale just as the other process parameters (temperature and time) did not vary, indicating uniform pretreatment of all straw batches. As only the solid fraction is used in the fermentation process at the Inbicon plant [19], sugars in the liquid fraction were not included in this study.

### Fractionation after pilot-scale pretreatment

After collection of the pretreated straw from the pilot plant, straw batches were washed in water 1:2 (vol/vol) to imitate the pilot-scale process wherein a washing step eliminates inhibitory soluble substances created during the pretreatment. Washed pretreated straw was pressed by hand with a towel, and 30 g of wet straw was separated into particle-size fractions of >1.2 mm, 0.63-1.2 mm and 50-630 μm by wet sieving. The fractions were pressed by hand with a towel, and the dry matter content was determined on a Sartorius MA30 (Sartorius AG, Germany) dry weight balance.

### Effect of enzyme loading on sugar yield of pilot-scale pretreated straw

Investigation of enzyme loading on sugar yield of the pilot-scale pretreated straw was performed in 100-ml plastic flasks with unfractionated pretreated and washed straw batches at 20% solid loadings in 50 mM Na-citrate buffer, pH 4.8, at three levels of enzyme loadings: 2.5, 5 and 10 FPU g^-1 ^dm pretreated straw by a 5:1 weight to weight (wt/wt) enzyme mix of cellulase (Celluclast, Novozymes, Bagsvaerd, Denmark) and cellobiase (Novozyme 188, Novozymes, Bagsvaerd, Denmark). Hydrolysis was performed according to the method described by Kristensen *et al*. [22], where enzymes were added immediately before incubation in a cement mixer at 50°C. After 120 h, flasks were boiled for 10 min to inactivate enzymes and 2-ml aliquots were removed, filtered and analyzed for glucose and xylose by HPLC. All treatments were carried out in triplicate.

### Effect of particle size of pilot-scale pretreated straw on sugar yield

Investigation of sugar yield of the different particle-size fractions was performed in 50-ml glass flasks with the three particle-size separated fractions (coarse, medium and small) and unfractionated straw at 5% solid loadings in 75 mM Na-citrate buffer, pH 4.8, at 5 FPU g^-1 ^dm pretreated straw by a 5:1 (wt/wt) enzyme mix of cellulase (Celluclast, Novozymes) and cellobiase (Novozyme 188, Novozymes). Flasks were incubated at 50°C and 150 rpm. After 116 h, 2-ml aliquots were removed, boiled for 10 min to inactivate enzymes and filtered to be analyzed for glucose and xylose by HPLC. All treatments were carried out in triplicate.

### Effect of particle size prior to pretreatment on sugar yield

To test whether particle-size distribution before pretreatment had the same effect on sugar yield as after pretreatment, we fractionated a single straw sample prior to pretreatment. Raw straw was taken from the Ambition bale from Holstebro and fractionated on a series of sieves after milling on a 1-mm screen, resulting in particle-size fractions of 425-850 μm, 250-425 μm, 180-250 μm and <180 μm. Pretreatment and hydrolysis of these fractions as well as that of an unfractionated sample were done in a 96-well steel plate as described by Studer *et al*. [23] at 1% solid loading and an enzyme loading of 60 FPU g^-1 ^glucan and xylan in raw material of a 5:1 (wt/wt) enzyme mix of cellulase (Celluclast, Novozymes) and cellobiase (Novozyme 188, Novozymes). This was achieved by loading 2.5 mg dm material per well, adding deionized water to a total volume of 250 mg and soaking for 4 h before heating to 180°C for 17.6 min, severity index 3.6 [21]. Then 12.5 μL of 1 M Na-citrate buffer, 2.5 μL of 1 g L^-1 ^NaN_3_, and 13 μL of diluted enzyme mix (diluted 10 times with 50 mM citric acid buffer, pH 4.8) were added to each well. Hydrolysis ran for 72 h at 50°C and 150 rpm. Sugar concentrations in each well were analyzed by HPLC. All treatments were done in triplicate by running three plates.

## Results and Discussion

### Effect of pilot-scale pretreatment on cultivars

Chemical properties of nonpretreated and pretreated straw are given in Table 1. Statistically, there was no difference in the composition of the nonpretreated cultivars. Compositional analysis before pretreatment therefore did not point to any cultivars with a higher saccharification potential.

**Table 1:** Chemical properties^a^

		Composition before pretreatment				Composition after pretreatment			
**Cultivar**	**Site**	**Cell** **(%)**	**Hemicell ** **(%)**	**Lignin** **(%)**	**Ash** **(%)**	**Cell** **(%)**	**Hemicell ** **(%)**	**Lignin** **(%)**	**Ash ** **(%)**

Ambition	H	37.1 (0.3)	27.6 (0.1)	20.9 (0.7)	3.7 (0.0)	57.3 (0.6)	5.8 (0.1)	27.6 (1.7)	3.4 (0.1)
Hereford	H	35.6 (0.1)	27.6 (0.1)	19.9 (0.2)	4.7 (0.0)	55.6 (0.7)	7.9 (0.1)	26.2 (1.2)	4.6 (0.2)
Skalmeje	H	36.4 (0.2)	27.2 (0.1)	19.9 (0.2)	5.2 (0.0)	54.8 (0.0)	7.3 (0.1)	24.3 (0.8)	8.5 (0.3)
Smuggler	H	36.6 (1.2)	28.4 (0.8)	19.3 (0.1)	4.2 (0.0)	58.6 (0.6)	6.9 (0.0)	26.4 (0.5)	4.0 (0.0)
Frument	H	35.9 (1.4)	27.0 (1.0)	20.1 (0.2)	4.6 (0.0)	58.9 (1.4)	6.7 (0.0)	27.0 (1.1)	2.6 (0.1)
Ambition	F	35.7 (0.3)	24.6 (0.2)	19.6 (0.3)	6.9 (0.1)	60.2 (0.4)	6.3 (0.1)	27.5 (0.2)	4.3 (0.1)
Hereford	F	36.2 (0.1)	25.5 (0.1)	20.3 (0.5)	3.8 (0.1)	59.8 (3.8)	7.5 (0.5)	27.0 (0.1)	4.4 (0.0)
Skalmeje	F	36.8 (0.8)	25.2 (0.3)	19.8 (0.1)	6.6 (0.0)	56.2 (0.2)	7.3 (0.1)	23.7 (0.3)	9.4 (0.1)
Smuggler	F	36.5 (0.3)	25.1 (0.2)	20.2 (0.3)	3.9 (0.1)	58.5 (0.4)	5.1 (0.0)	28.5 (0.4)	4.3 (0.1)
Frument	F	37.0 (0.4)	25.9 (0.7)	20.1 (0.3)	3.9 (0.0)	59.0 (0.5)	5.9 (0.0)	27.4 (0.8)	3.6 (0.0)

Variation	H, F	0.27	1.66	0.18	1.37	3.33	1.02	2.29	4.90
Variation	H	0.33	0.28	0.31	0.32	3.29	0.97	1.58	5.18
Variation	F	0.27	0.26	0.09	2.58	2.41	0.99	3.40	5.64

^a^Chemical properties of five cultivars of wheat straw from two sites (H = Holstebro and F = Fynen) before and after hydrothermal pretreatment (195°C, 10 min). Values are averages (*n *= 3), and standard deviations are given in parentheses. Cell = cellulose, Hemicell = hemicellulose. Variation in biochemical compounds within materials from both sites and the individual sites are presented at the bottom.

After pretreatment, lignin (*P *= 0.0324) and ash content (*P *= 0.0004) varied between cultivars. This was due to Skalmeje displaying low lignin content after pretreatment which was significantly different from lignin-rich pretreated Smuggler and Ambition and having higher ash content than all other batches (Table 1). Thus, wheat cultivars respond differently to hydrothermal pretreatment in a way that introduces chemical differences, resulting in larger variations in the composition of pretreated straw than in nonpretreated straw (Table 1). Skalmeje is known for high straw stiffness and good pest resistance. Straw stiffness may be associated with modified anatomical features of the stems and changed chemical characteristics of the cell walls, which may decrease the degradability of the straw [24] as seen for Skalmeje.

### Effect of enzyme loading on sugar yield from different cultivars

**Figure 1:**
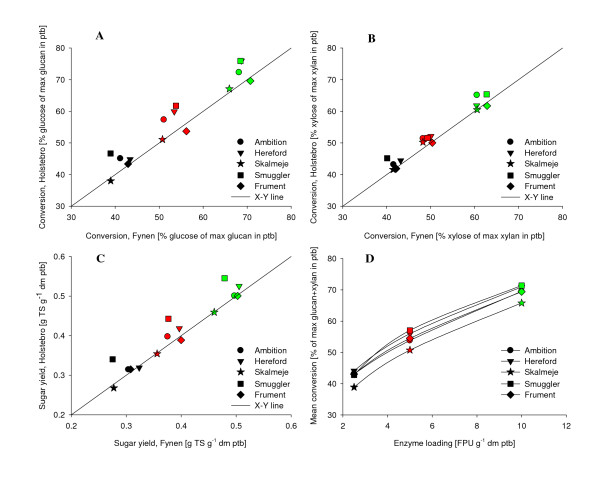
**Sugar yield at different enzyme loadings**. Sugar yield from hydrolysis of pretreated straw from five cultivars from two sites (Holstebro and Fynen) at varying enzyme loadings (black symbols = 2.5 FPU, red symbols = 5 FPU, green symbols = 10 FPU g^-1 ^dm pretreated straw) presented as **(A) **glucose conversion as a percentage of maximum glucan in pretreated biomass (ptb), **(B) **xylose conversion as a percentage of maximum xylan in pretreated biomass, **(C) **total sugar (TS) yield in gram per gram dm pretreated biomass and **(D) **average of total sugar convertibility from the two sites as a percentage of maximum glucan and xylan as a function of different enzyme loading.

The results of hydrolysis at different enzyme loading levels are given in Figure 1. Data are presented as the convertibility of cellulose (Figure 1A) and xylan (Figure 1B) from each cultivar grown at two sites and as yield of total sugars per gram dry matter of pretreated biomass used in the hydrolysis (Figure 1C). In these plots, three clusters of increasing yield arise from different levels of enzyme loading. Generally, 45% of the cellulose and xylan available in pretreated straw was converted into sugar at 2.5 FPU g^-1 ^dm ptb, whereas 55% was converted at 5 FPU g^-1 ^dm ptb and 70% was converted at 10 FPU g^-1 ^dm ptb. Thomsen *et al*. [25] obtained similar maximum cellulose convertibility of wheat straw (72% of pretreated solid fraction) at higher enzyme loading (30 FPU g^-1 ^dm, 2% dm solid). This was probably due to the use of a considerably lower pretreatment severity (R_0 _= 1.6) than in our experiment. Prehydrolysis and simultaneous saccharification and fermentation (10% dm solid) of similarly pretreated wheat straw conducted at 15 FPU g^-1 ^dm with an enzyme mixture comparable to ours resulted in approximately 85% cellulose conversion after 144 h [20]. As intended, we stayed below maximum conversion level so that potential genotypic differences could be visible.

There was a trend toward larger conversions of straw grown at Holstebro compared to Fynen (Figures 1, as most cultivars were plotted above the *x-y *line. When comparing results from genotypes at different enzyme loadings (Figure 1D), Skalmeje had noticeably lower convertibility than any other cultivars, whereas Hereford and Smuggler seemed to have a higher convertibility at all enzyme levels. The poor result of Skalmeje was particularly interesting, considering that Skalmeje had a low content of cellulose after pretreatment (Table 1), whereby enzyme loading was 4-7% higher for Skalmeje than the most cellulose-rich cultivar, Ambition, when assessed in FPU g^-1 ^cellulose in pretreated material. A mixed-effects model was used to test effects of cultivar and enzyme loading on total sugar yield with batch and site as random variables (Table 2, model A).

**Table 2:** Analysis of variance in total sugar yield^a^

Sugar yield (ANOVA): Model A					
	**Df num**	**Df den**	**F value**	**Pr > F**	**Significance**
Enzyme loading	2	68	8486.04	< 0.0001	***
Cultivar	4	68	2.10	0.0907	n.s.
Enzyme loading × cultivar	8	68	4.66	0.0001	***

**Sugar yield (ANOVA): Model B**					

	**Df num**	**Df den**	**F value**	**Pr > F**	**Significance**
Cultivar	4	20	9.01	0.0002	***
					
	**Mean sugar yield (g g **^**-1 **^**ptb)**				
Hereford	0.292^a^				
Ambition	0.287^ab^				
Smuggler	0.282^ab^				
Frument	0.272^b^				
Skalmeje	0.249^c^				

**Sugar yield (ANOVA): Model C**					

	**Df**	**Sum sq**	**Mean Sq**	**Pr > F**	**Significance**
Batch	9	0.019	0.0022	< 0.0001	***
Size	2	0.023	0.011	< 0.0001	***
Batch × size	18	0.00086	0.000048	0.2831	n.s.
Residual	59	0.0023	0.000039		
					
	**Mean sugar yield (g g **^**-1 **^**dm ptb)**				
Small	0.295^a^				
Medium	0.268^b^				
Coarse	0.257^c^				

^a^Analysis of variance (ANOVA) of total sugar yield (g g ^-1 ^dm pretreated biomass) affected by cultivar and enzyme loading in model A, by cultivar from total fractions of the second hydrolysis in model B and by batch, size and interaction from coarse, medium and small fraction in model C. Mean sugar yield (n = 3) with different numbers are significantly different (*P *≥ 0.05). Significance values: *P = 0.05, **P = 0.01 and ***P = 0.001; n.s. = not significant.

**Figure 2:**
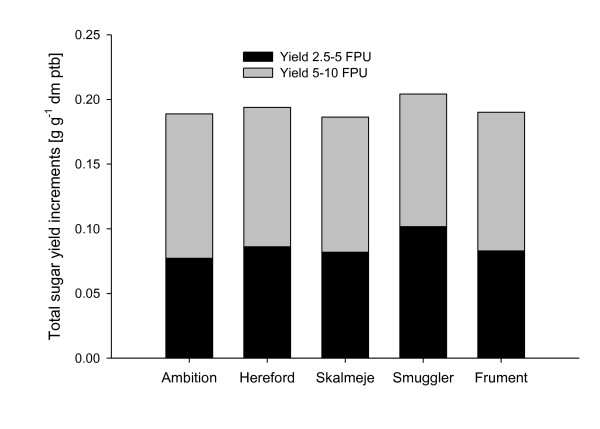
**Total sugar yield increments with increasing enzyme concentration**. Analysis of variance estimates of total sugar yield increments in g g^-1 ^dm pretreated biomass from each cultivar when increasing the enzyme concentration from 2.5 to 5 FPU g^-1 ^dm pretreated biomass and from 5 to 10 FPU g^-1 ^dm pretreated biomass.

As expected, enzyme loading had a significant effect on sugar yield. Cultivar was not significant, but there was a significant interaction between enzyme loading and cultivar (Table 2, model A), that is, the response to enzyme loading depended on the cultivar. The means of the interaction display the increment in sugar yield with increasing enzyme level for each cultivar as shown in Figure 2. Skalmeje had the lowest overall response to increased enzyme loading, while Smuggler had the overall highest effect of the increased enzyme concentration, especially at low loadings (2.5 to 5 FPU). Ambition had the lowest response of enzymes until 5 FPU, but had the most efficient use of enzyme loading from 5 to 10 FPU. Hence, the cultivars respond differently in terms of sugar yield when the enzyme loading is varied.

In the second hydrolysis experiment where enzyme loading was fixed at 5 FPU g^-1 ^dm ptb, the effect of cultivar was significant (Table 2, Model B) with mean sugar yields in decreasing order for Hereford > Ambition > Smuggler > Frument > Skalmeje. The convertibility of glucan plus xylan (% TS) of Hereford exceeded that of Skalmeje by 13%. Ambition, Smuggler and Frument converted 12%, 10% and 6% more of their maximum available glucan and xylan, respectively, than Skalmeje. Differences between cultivars based on weight are even more pronounced, where Hereford straw released 17% more sugar than Skalmeje straw (0.25 g TS g^-1 ^dm pretreated biomass); Ambition released 15% more, Smuggler released 13% more and Frument released 9% more sugar than Skalmeje.

Schell *et al*. [26] published data on pilot-scale variability on replicate runs of biomass, using corn stover and pretreatment conditions of 165°C, 8 min, 1.4% (wt/wt) acid concentration. Over six replicate runs, they reached a standard deviation between 5% and 20% of the average values in xylose and furfural yields, cellulose conversions and carbon mass balance results. The authors list uncertainties in residence time calibration and changes in feedstock acid neutralizing capacity as possible factors [26]. Thus, we have reason to believe that the pilot plant process influences the specific results of the genotypes presented here. Further investigations are needed to separate the variation caused by cultivar and process conditions during pilot-scale pretreatment. However, our data indicate that further attention to breeding for high-sugar-yielding straw cultivars under modest enzyme loading could be warranted.

### Effect of particle size on sugar yield

**Figure 3:**
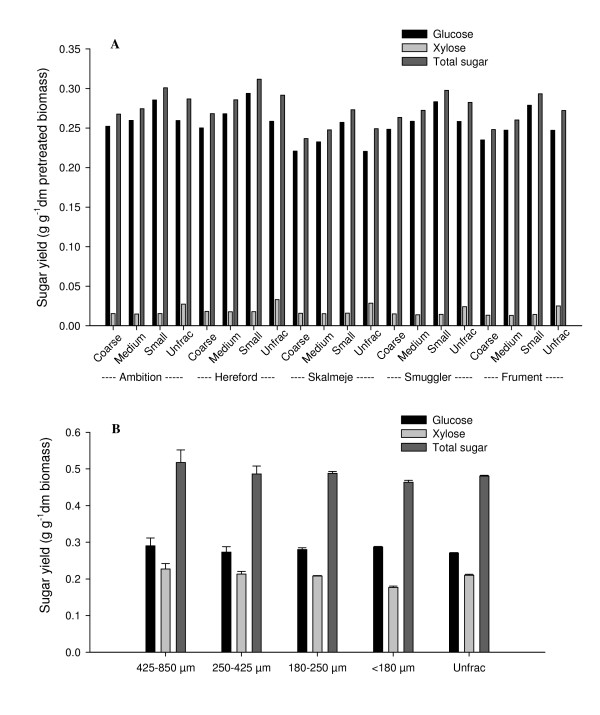
**Sugar yield from particle-size fractionations constructed after or before pretreatment**. **(A) **Yield of glucose, xylose and total sugar in coarse, medium, small fractions and unfractionated samples (Unfrac) of pilot-scale pretreated straw from five cultivars averaged from two sites. **(B) **Yield of glucose, xylose and total sugar in four particle-size fractions and one unfractionated sample of milled, sieved and bench-scale pretreated and hydrolyzed wheat straw from the cultivar Ambition. Error bars are standard deviations over triplicate samples.

The sugar yield from different particle-size fractions, coarse (> 1.2 mm), medium (0.63-1.2 mm) and small (50-630 μm), as well as the yield from unfractionated samples (Unfrac), are presented in Figure 3A. We found only little xylan conversion in unfractionated samples owing to the severe hemicellulose removal during pretreatment (Table 1), and even less was found in the size fractions owing to extended washing in the fractionation process. Separate hydrolysis of small particles resulted very consistently in 5% to 10% higher sugar yield than hydrolysis of unfractionated samples (Figure 3A). In contrast, separate hydrolysis of coarse fractions resulted in approximately 10% lower sugar yield than hydrolysis of unfractionated samples. Total sugar yield from the separately hydrolyzed fractions was significant depending on particle size (Table 2, model C) with sugar yield from small particle fractions (averaged 0.29 g TS g^-1 ^dm ptb; Figure 3A) being 11% to 21% higher than sugar yield from coarse particle fractions (averaged 0.24 g TS g^-1 ^dm ptb; Figure 3A). Presumably, when the size of the substrate particle is reduced, the accessible surface for enzymatic attack is increased and the lengths of entry and exit paths for enzymes and hydrolysis products, respectively, are reduced [27,28]. To our knowledge, this is the first study comparing convertibility of particle-size fractions produced by pilot-scale pretreatment.

Several studies have fractionated straw into size fractions by milling and separation before pretreatment and found smaller particles to have the same total sugar yield and hydrolysis rate as the larger particles after pretreatment [16,29]. In line with this, we found equal total sugar yields (*P *= 0.0594) regardless of particle sizes when fractionation was done before pretreatment (Figure 3B). Xylan conversion decreased with decreasing particle size (*P *= 0.0002), whereas the cellulose conversion stayed the same (*P *= 0.2634). Xylose yield was considerable at bench scale (fractionation before pretreatment and no washing of solid material before hydrolysis; Figure 3B) compared with xylose yield at pilot-scale (fractionation after pretreatment and washing before hydrolysis; Figure 3A). Thus our results are consistent with those of Pedersen and Meyer [16], who found total yield of monomers of wheat straw fractions to be similar after pretreatment as a result of xylose yield counteracting an increase in glucose yield with reduction of particle sizes.

Zeng *et al*. [29] concluded that the difference between particle-size yields is eliminated when corn stover is pretreated, because pores and hollows are made in the larger particles during pretreatment, rendering them more susceptible to conversion. However, on the basis of our results, we conclude that the pretreatment fractionates the biomass and induces differences in total sugar yield from particle-size fractions except when xylose conversion is a major factor.

### The effect of particle-size distributions and chemical composition on sugar yield

**Figure 4:**
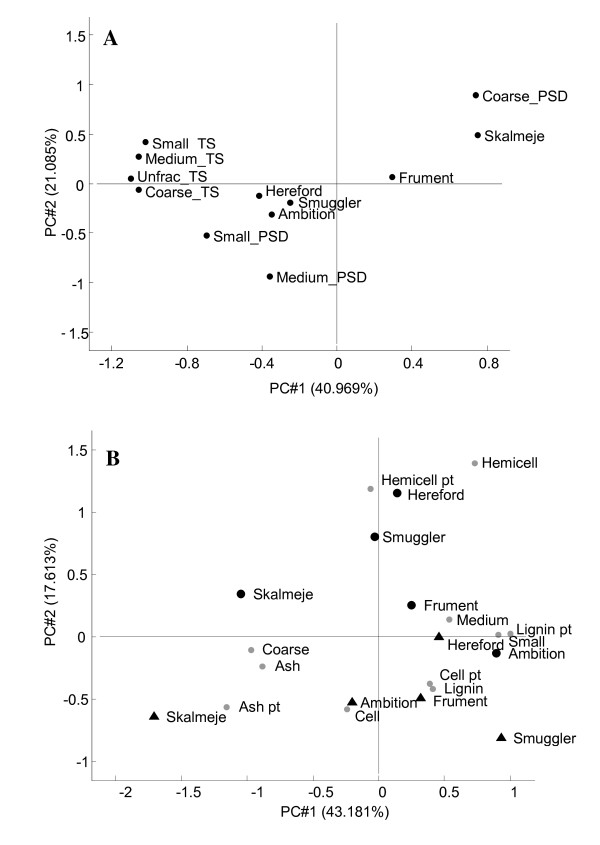
**Plot of sugar conversion and particle size distribution and biplot of sugar yield and chemical changes during pretreatment**. **(A) **Loadings along the first two principal components obtained in a principal component analysis model including total sugar (TS) conversions of unfractionated (Unfrac), coarse, medium and small fractions and particle-size distribution (PSD) of coarse, medium and small fractions as percentage of the total mass. Placing of straw cultivars is based on samples from both sites. **(B) **Scores (straw cultivars) and loadings along the first two principal components obtained in a partial least squares (PLS) regression analysis model of total sugar yield in unfractionated samples correlated with chemical composition (Cell = cellulose, Hemicell = hemicellulose) before and after pretreatment (pt) as a percentage of total mass and particle size distribution of coarse, medium and small fractions as a percentage of total mass. Cultivars marked with circles were grown at Holstebro, and cultivars marked with triangles were grown at Fynen.

Differences in total sugar yield between cultivars (Table 2, Model B) can be attributed to either particle-size distribution in pretreated material (that is, if a cultivar results in many coarse particles during pretreatment, the overall yield will be poor) or a different conversion of particle-size fractions related to a certain cultivar. Figure 4A shows a PCA of total sugar (TS) conversion in the unfractionated samples and size-separated fractions related to particle-size distributions (PSD) and chemical composition. Loadings along the first two principal components obtained in the PCA indicate that Skalmeje is the only cultivar in which the mass of the coarse fraction is highly influential and negatively correlated to the sugar conversion in the unfractionated samples. Predictions of the sugar conversion in the unfractionated samples using a PLS calibration based on both particle-size distributions and conversions of the fractions can account for 96.4% of the variance (RMSE = 0.44), whereas predictions based solely on conversions explain 91.7% of the variance (RMSE = 0.67) and particle-size distribution alone explains 6.5% of the variance (RMSE = 2.64). This suggests that the differences between cultivars in sugar conversions of unfractionated samples resulted mainly from differences in the convertibility of the size fractions rather than from differences in particle-size distribution. The ability of the particle-size distribution to predict sugar conversion of unfractionated samples became even worse when Skalmeje data was removed from the data set. Most likely the substantial coarse fraction in Skalmeje straw is caused by the high straw stiffness of this cultivar.

To study the interaction of chemical parameters, a PLS calibration relating sugar yield in unfractionated samples with chemical composition before and after pretreatment to the particle-size distribution of the pretreated biomass was done (Figure 4B). Straw from the two sites was separated along the second principal component, suggesting that sugar yield from straw grown in Holstebro was more influenced than straw grown in Fynen by the amount of hemicellulose, probably related to higher removal of hemicellulose in Holstebro cultivars during pretreatment (Table 1). Skalmeje on both sites were separated from the other cultivars along the first principal component owing to higher ash content before and after pretreatment and larger coarse fraction. Although Skalmeje was the cultivar with lowest lignin content after pretreatment (Table 1), the primary cause for Skalmeje ending up with a different composition compared to the other cultivars was the change in ash content after pretreatment (Figure 4B). In summary, the variability in sugar yield between cultivars depended not on differences in analyzed chemical composition of raw material, but rather on differences in the removal of hemicellulose, accumulation of ash and (for Skalmeje) particle-size distribution introduced by the pretreatment.

## Conclusions

When comparing total sugar yield from a pilot-scale pretreatment of five commercially grown wheat straw cultivars grown at two different sites, the cultivars did indeed show different yields. Depending on experimental conditions, the effect of cultivar was either highly significant or the interaction between cultivar and enzyme loading was significant. This indicates that the optimal process parameters depend on the cultivar, just as the potential of breeding for cultivars with a higher processability to fermentable sugars is confirmed.

Sugar yield from separately hydrolyzed particle-size fractions separated after pretreatment of each cultivar showed that finer particles had higher yield than coarse particles. Particle-size distributions were found to affect total sugar conversions only in the most recalcitrant cultivar. High ash content and a large fraction of coarse particles were negatively correlated with total sugar conversion. We conclude that genetic variability in sugar yield exists for wheat straw when processed under large pilot-scale conditions.

## Competing interests

The authors declare that they have no competing interests.

## Authors' contributions

JL coordinated collection of materials and performed pretreatment at pilot scale and small scale, fractionation and hydrolysis experiments, statistical analysis, data interpretation, and drafting and completion of the manuscript. SB participated in the design of the study, statistical analysis, data interpretation and drafting of the manuscript. HJ participated in the design of the study and data interpretation. CF conceived the study, contributed to intellectual discussions and thoroughly reviewed the manuscript. JM participated in the design of the study and data interpretation. All authors read and approved the final manuscript.
